# UHRF1 downregulation promotes T follicular helper cell differentiation by increasing BCL6 expression in SLE

**DOI:** 10.1186/s13148-021-01007-7

**Published:** 2021-02-10

**Authors:** Limin Liu, Longyuan Hu, Linxuan Yang, Sujie Jia, Pei Du, Xiaoli Min, Jiali Wu, Haijing Wu, Hai Long, Qianjin Lu, Ming Zhao

**Affiliations:** 1 Department of Dermatology, Second Xiangya Hospital, Central South University, #139 Renmin Middle Road, Changsha, 410011 Hunan China; 2 Research Unit of Key Technologies of Diagnosis and Treatment for Immune-Related Skin Diseases, Chinese Academy of Medical Sciences (2019RU027), Changsha, Hunan China; 3 Department of Pharmacy, Central South University, The Third Xiangya Hospital, Changsha, China

**Keywords:** Systemic lupus erythematosus, BCL6, Tfh cells, UHRF1, Epigenetics

## Abstract

**Background:**

Transcription factor B cell lymphoma 6 (BCL6) is a master regulator of T follicular helper (Tfh) cells, which play a crucial role in the pathogenesis of systemic lupus erythematosus (SLE). However, the mechanisms by which BCL6 expression is regulated are poorly understood. Ubiquitin-like with PHD and RING finger domains 1 (UHRF1) is an important epigenetic factor that regulates DNA methylation and histone modifications. In the present study, we assessed whether UHRF1 can regulate BCL6 expression and influence the differentiation and proliferation of Tfh cells.

**Results:**

Compared to healthy controls, the mean fluorescence intensity of UHRF1 (UHRF1-MFI) in Tfh cells from SLE patients was significantly downregulated, whereas that of BCL6 (BCL6-MFI) was significantly upregulated. In vitro, UHRF1 knockdown led to BCL6 overexpression and promoted Tfh cell differentiation. In contrast, UHRF1 overexpression led to BCL6 downregulation and decreased Tfh cell differentiation. In vivo, conditional UHRF1 gene knockout (UHRF1-cKO) in mouse T cells revealed that UHRF1 depletion can enhance the proportion of Tfh cells and induce an augmented GC reaction in mice treated with NP-keyhole limpet hemocyanin (NP-KLH). Mechanistically, UHRF1 downregulation can decrease DNA methylation and H3K27 trimethylation (H3K27me3) levels in the *BCL*6 promoter region of Tfh cells.

**Conclusions:**

Our results demonstrated that UHRF1 downregulation leads to increased BCL6 expression by decreasing DNA methylation and H3K27me3 levels, promoting Tfh cell differentiation in vitro and in vivo. This finding reveals the role of UHRF1 in regulating Tfh cell differentiation and provides a potential target for SLE therapy.

## Background

Systemic lupus erythematosus (SLE) is a systemic autoimmune disease that primarily affects women of gestational age and damages various tissues and organs [1–3]. Several factors, including genetic susceptibility, environment, hormones and epigenetic factors contribute to SLE [2, 4–7]. Abnormally activated and proliferated CD4^+^T cells play an important role in the pathogenesis of SLE [8, 9].

T follicular helper (Tfh) cells, which were discovered in 2000, are members of the CD4^+^ T cell family. The maturation and differentiation of Tfh cells occurs in germinal centres (GCs) [10, 11]. Tfh cells promote GC reaction by inducing the biological activities of B cells, such as affinity maturation, antibody isotype conversion, plasma cell differentiation and memory B cells production [12, 13]. Tfh cells express a number of surface markers, such as CXC-chemokine receptor 5 (CXCR5), programmed cell death-1 (PD1) and inducible T-cell co-stimulator (ICOS). Transcription factor BCL6, which is considered to be a master regulator of Tfh cells, controls Tfh cell differentiation and function [14]. Increasing evidence as shown that the proportion of Tfh cells is increased in SLE patients, indicating that they play a crucial role in the dysregulated antibody responses associated with SLE [15–17]. However, the mechanisms by which the aberrant differentiation of Tfh cells is regulated remain poorly understood.

Epigenetics refers to inheritable changes in the chromosome that do not alter the DNA sequence and have a regulatory function in the development and differentiation of immune cells [18–20]. We previously showed that aberrant epigenetic modifications were associated with the differentiation of CD4^+^T cells in SLE. For example, reduced RFX1 expression can lead to IL-17A overexpression by decreasing DNA methylation and histone H3K9 trimethylation and increasing H3 acetylation [21]. In addition, MBD4 was shown to inhibit CD70 expression and enhance DNA methylation level of CD70 gene [22], while E4BP4 was observed to inhibit Tfh cell differentiation and BCL6 transcription by recruiting the repressive epigenetic modifiers HDAC1 and EZH2 [23, 24]. UHRF1 typically represses transcription by regulating DNA methylation or histone modification in the promoter of target genes. A number of previous studies showed that UHRF1 regulates the epigenetic modification of proto-oncogenes in various cancers [25–27]. However, the role of UHRF1 in the immune system and immune system diseases is only starting to be understood. UHRF1 can regulate the methylation level of the TNF-α gene by recruiting DNMT1 to enhance the secretion of proinflammatory factors in macrophages [28]. UHRF1 may be involved in the mechanism associated with the TGF-β-mediated differentiation of the induced Treg cells in inflammatory diseases [29].

In the present study, we assessed whether UHRF1 can regulate BCL6 expression and affect the differentiation and proliferation of Tfh cells. Our results demonstrated that UHRF1 knockdown can upregulate BCL6 expression and promote the differentiation of Tfh cells, suggesting that UHRF1 may play an important role in the pathogenesis of SLE.

## Results

### UHRF1 expression is reduced in circulating follicular helper T cells isolated from peripheral blood mononuclear cells (PBMCs) of SLE patients

**Fig. 1:**
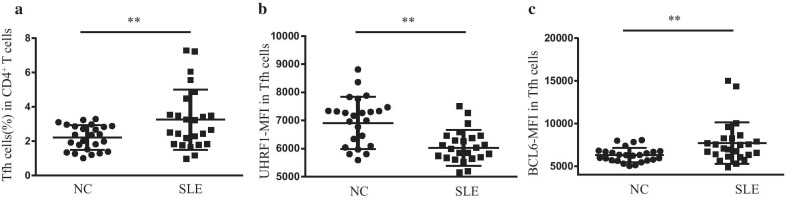
Comparison of UHRF1 expression in Tfh cells from PBMCs between SLE patients and healthy controls. (**a**–**c**) The proportion of Tfh cells, where the MFI of UHRF1 and BCL6 in Tfh cells from SLE patients (*n* = 25) and healthy controls (*n* = 25) was detected by flow cytometry. *P < 0.05, **P < 0.01 relative to control

We first measured CD4, PD1, CXCR5 expression in PBMCs by flow cytometry (FCM), the results of which showed that the Tfh cell proportion was significantly higher in SLE patients than healthy controls (Fig. 1a), consistent with our previous findings[30]. To compare the levels of UHRF1 expression in Tfh cells between SLE patients and healthy controls, we measured the mean fluorescence intensity (MFI) of UHRF1 (UHRF1-MFI) in Tfh cells by FCM. The results showed that the UHRF1-MFI was significantly lower in Tfh cells isolated from SLE patients than in those isolated from healthy controls (Fig. 1b). Furthermore, we measured the BCL6-MFI in Tfh cells, one of the most important transcription factors for the differentiation and function of Tfh cells. The results showed that the BCL6-MFI was significantly higher in Tfh cells isolated from SLE patients than in those from healthy controls (Fig. 1c). These data indicated that the reduced expression of UHRF1 in Tfh cells may be functionally involved in SLE.

### *UHRF1 knockdown upregulates BCL6 expression and promotes Tfh cell differentiation *in vitro

**Fig. 2:**
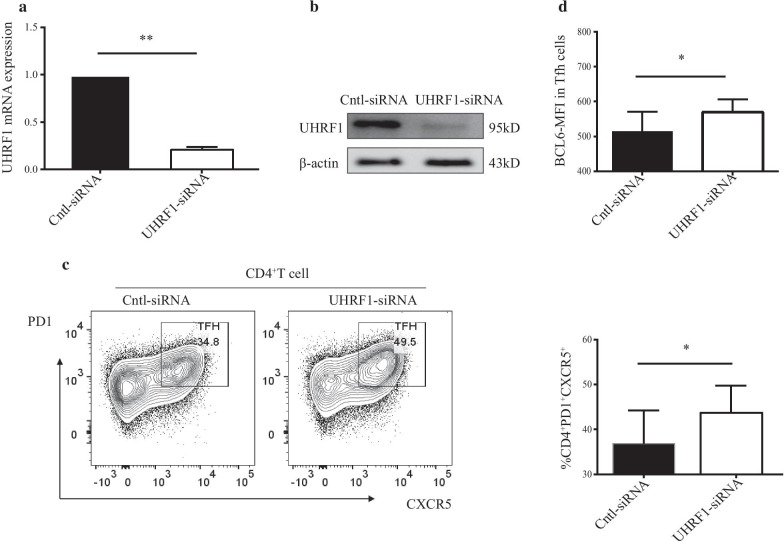
Knockdown of UHRF1 upregulates BCL6 expression and promotes Tfh cell differentiation in vitro. (**a**, **b**) The RT-qPCR results (**a**) and western blot results (**b**) showed that UHRF1 mRNA and protein levels were increased in the transfected cells with UHRF1-siRNA compared to the negative control. (**c**, **d**) Flow cytometry analysis showing that the percentage of Tfh cells (c) and the MFI of BCL6 protein (**d**) were increased in the transfected cells with UHRF1-siRNA compared to the negative control. The values are the averages of at least three biological replicates, and all data shown are the means ± SD. *P < 0.05, **P < 0.01 relative to control

We next investigated whether UHRF1 is involved in regulating BCL6 expression in Tfh cells. To this end, we knocked down UHRF1 expression via RNA interference (RNAi) in naïve CD4^+^T cells isolated from the PBMCs of healthy volunteers by transfecting them with UHRF1-siRNA or Cntl-siRNA. Subsequently, the transfected cells were cultured for 48 h under Tfh cell-polarizing conditions. The transfected cells were used to assess the changes in UHRF1 mRNA and protein expression by RT-qPCR and western blot analysis (Fig. 2a, b, Additional file 4). The FCM results showed that the proportion of Tfh cells (CD4^+^CXCR5^+^PD-1^+^) and the BCL6-MFI were increased in the group transfected with UHRF1-siRNA compared to the control group, indicating that UHRF1 knockdown promotes Tfh cell differentiation (Fig. 2c, d).

### *UHRF1 overexpression downregulates BCL6 expression and reduces Tfh cell differentiation *in vitro

**Fig. 3:**
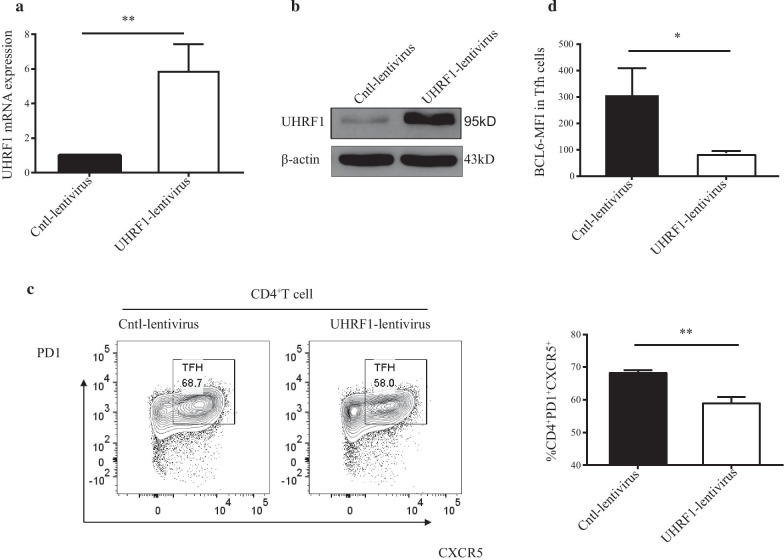
UHRF1 overexpression downregulates BCL6 expression and reduces Tfh cell differentiation. (**a**, **b**) Increased levels of UHRF1 mRNA (**a**) and protein (**b**) were observed in the cells transfected with UHRF1 overexpression compared to those with the empty control. (**c**, **d**) Flow cytometry results showed that the percentages of Tfh cells (c) and the MFI of BCL6 protein (**d**) were decreased in cells transfected with the UHRF1-lentivirus compared to the blank control. The values are the averages of at three biological replicates, and all data shown are the means ± SD. *P < 0.05, **P < 0.01 relative to control

We subsequently assessed whether UHRF1 upregulation can inhibit BCL6 expression in Tfh cells. We increased UHRF1 expression in naïve CD4^+^T cells isolated from the PBMCs of healthy volunteers by transfecting them with a UHRF1 lentiviral expression vector (UHRF1-lentivirus) or empty lentivirus (Cntl-lentivirus). The cells were cultured for 72 h post-transfection under Tfh cell-polarizing conditions. Then, UHRF1 expression in the harvested cells was assessed by RT-qPCR and western blot analysis. The results showed that UHRF1 mRNA and protein levels were significantly higher in cells transfected with UHRF1-lentivirus than those transfected with Cntl-lentivirus (Fig. 3a, b, Additional file 4). Furthermore, FCM results showed that the proportion of Tfh cells and BCL6 expression levels were significantly lower in cells transfected with UHRF1-lentivirus compared tan in the negative control (Fig. 3c, d).

### UHRF1 regulates the epigenetic modification of the BCL6 promoter region

**Fig. 4:**
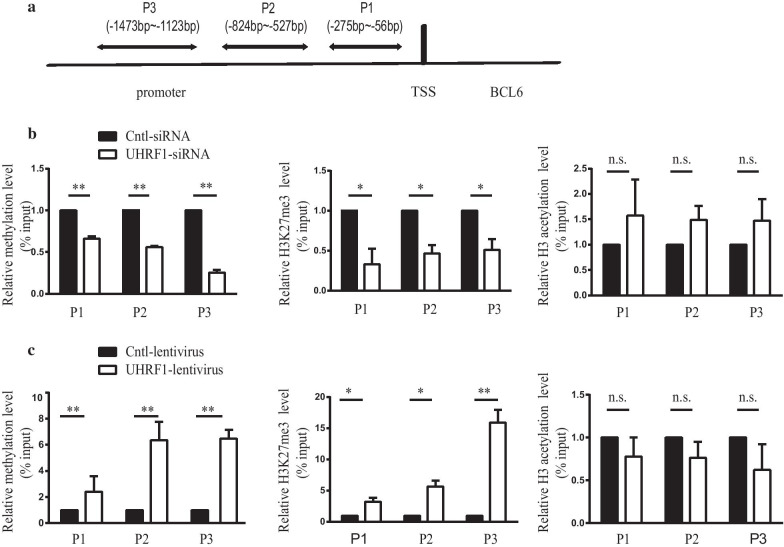
UHRF1 regulates the epigenetic modifications of the *BCL*6 promoter region. (**a**) The Schematic diagram showing the location of the P1, P2, and P3 regions in the *BCL6* promoter for MeDIP-qPCR and ChIP-qPCR. (**b**) The levels of DNA methylation and H3K27me3 were downregulated in the induced Tfh cells with UHRF1-siRNA compared to the negative control. No significant changes in H3 acetylation levels were observed. (**c**) The levels of DNA methylation and H3K27me3 were upregulated in the induced Tfh cells with the UHRF1-lentivirus compared to the negative control. No significant change in H3 acetylation levels were observed. The values are the averages of at three biological replicates, and all data shown are the means ± SD. *P < 0.05, **P < 0.01 relative to control

UHRF1 is an important epigenetic factor that can regulate DNA methylation and histone modification [24]. According to the results of previous studies, UHRF1 can interact with DNMT1 and EZH2 to form a regulatory complex to regulate DNA methylation and H3K27me3 of gene promoters [31, 32]. Therefore, we hypothesized that UHRF1 regulates BCL6 expression through epigenetic mechanisms. To test this possibility, we designed three primers pairs at the P1, P2, and P3 locations in the *BCL6* promoter for ChIP-qPCR and MeDIP-qPCR analyses (Fig. 4a). We assessed the level of DNA methylation, H3K27me3 and H3 acetylation in the induced Tfh cells with UHRF1 knockdown or overexpression. The MeDIP-qPCR and ChIP-qPCR results showed that the levels of DNA methylation and H3K27me3 in the *BCL6* gene promoter region were downregulated in the induced Tfh cells with UHRF1 knockdown compared to that observed in the negative control (Fig. 4b). No significant change in H3 acetylation levels were observed in the promoter region of the *BCL6* gene (Fig. 4b). In contrast, the levels of DNA methylation and H3K27me3 were upregulated, while no significant changes in H3 acetylation levels were observed in the promoter region of the *BCL6* gene in the induced Tfh cells with UHRF1 overexpression compared to the blank control (Fig. 4c).

### *UHRF1 deficiency increases the proportion of Tfh cells *in vivo* and promotes an augmented GC reaction stimulated by NP-KLH*

**Fig. 5:**
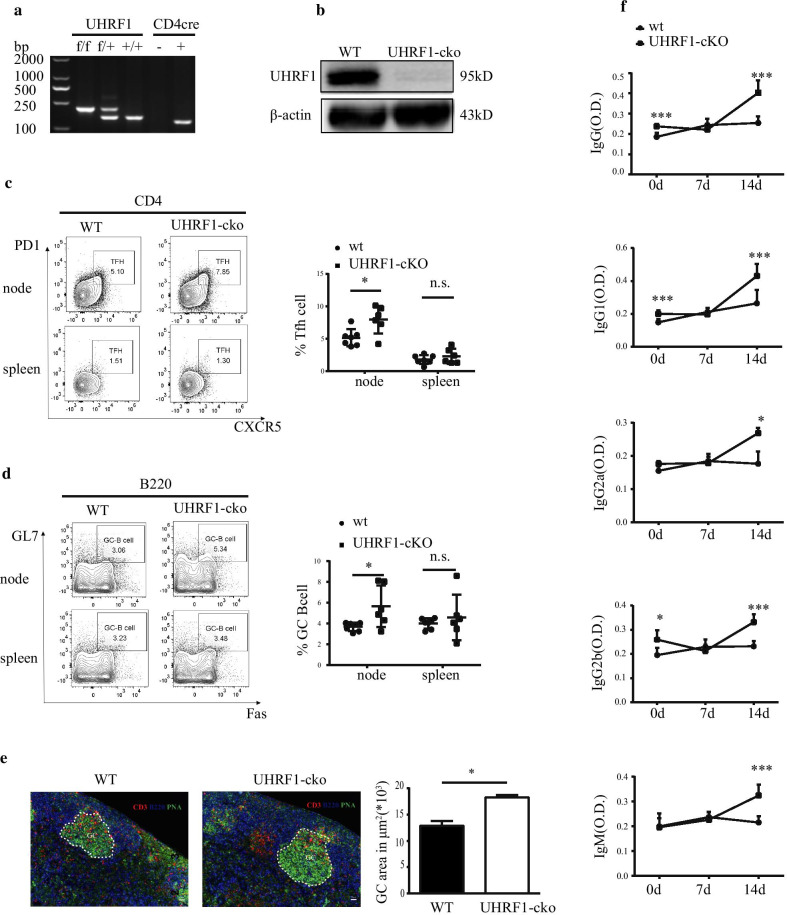
UHRF1 deficiency promotes GC responses induced by NP-KLH after immunization with NP-KLH in WT or UHRF1-cKO mice for 14 days. (**a**, **b**) The generation of UHRF1-cKO mice, representative gel of PCR identification and western blot analysis in CD4 ^+^ T cells for UHRF1 expression in UHRF1-cKO and WT mice. (**c**) Flow cytometry analysis of Tfh cell markers with CD4^+^CXCR5^+^PD1^+^. (**d**) Flow cytometry analysis of GC-B cell markers with B220^+^Fas^+^GL7^+^. (**e**) Immunofluorescence of GCs from WT and UHRF1-cKO mice, representative images of CD3, B220 and PNA staining of DLNs (bar, 50 µm). (**f**) Levels of serum specific total IgG, IgG1, IgG2a, IgG2b and IgM for NP-KLH immunization at days 0, 7 and 14 by ELISA. All data are shown as the means ± SD. *P < 0.05, **P < 0.01, ***P < 0.001 relative to controls

To investigate the role of UHRF1 in Tfh cells in mediating humoral immune responses in vivo, we first generated mice with a conditional knockout of the *UHRF1* gene in T cells (UHRF1-cKO) by crossing UHRF1^flox/flox^ mice with CD4-cre mice (Fig. 5a, Additional file 4). We isolated CD4^+^ T cells from the spleens of UHRF1-cKO and wild-type (WT) mice and assessed the expression of UHRF1. The western blot results indicated the UHRF1 expression was significantly repressed in CD4^+^ T cells from UHRF1-cKO mice (Fig. 5b, Additional file 4).

Subsequently, we immunized UHRF1-cKO and WT mice with NP-KLH antigen to and assessed the resulting proportions of Tfh cells and GC responses. Compared to the WT group, the proportions of Tfh cells and B220^+^Fas^+^GL7^+^ cells (GC B cells) were significantly increased in the draining lymph nodes (DLNs) of UHRF1-cKO mice based on the FCM results (Fig. 5c, d). Subsequently, PNA, B220 and CD3 staining of histological sections of DLNs revealed an enhanced GC response in UHRF1-cKO mice compared to that observed in WT mice (Fig. 5e). In addition, we also assessed the levels of total IgG, IgG1, IgG2a, IG2b and IgM in serum at day 0, day 7 and day 14 after NP-KLH immunization. The ELISA results showed that the titres of the various subtypes of antibodies were higher in the UHRF1-cKO mice than those observed in the WT mice (Fig. 5f). The above results indicated that UHRF1 deficiency promotes Tfh cell differentiation and GC responses in mice.

## Discussion

**Fig. 6:**
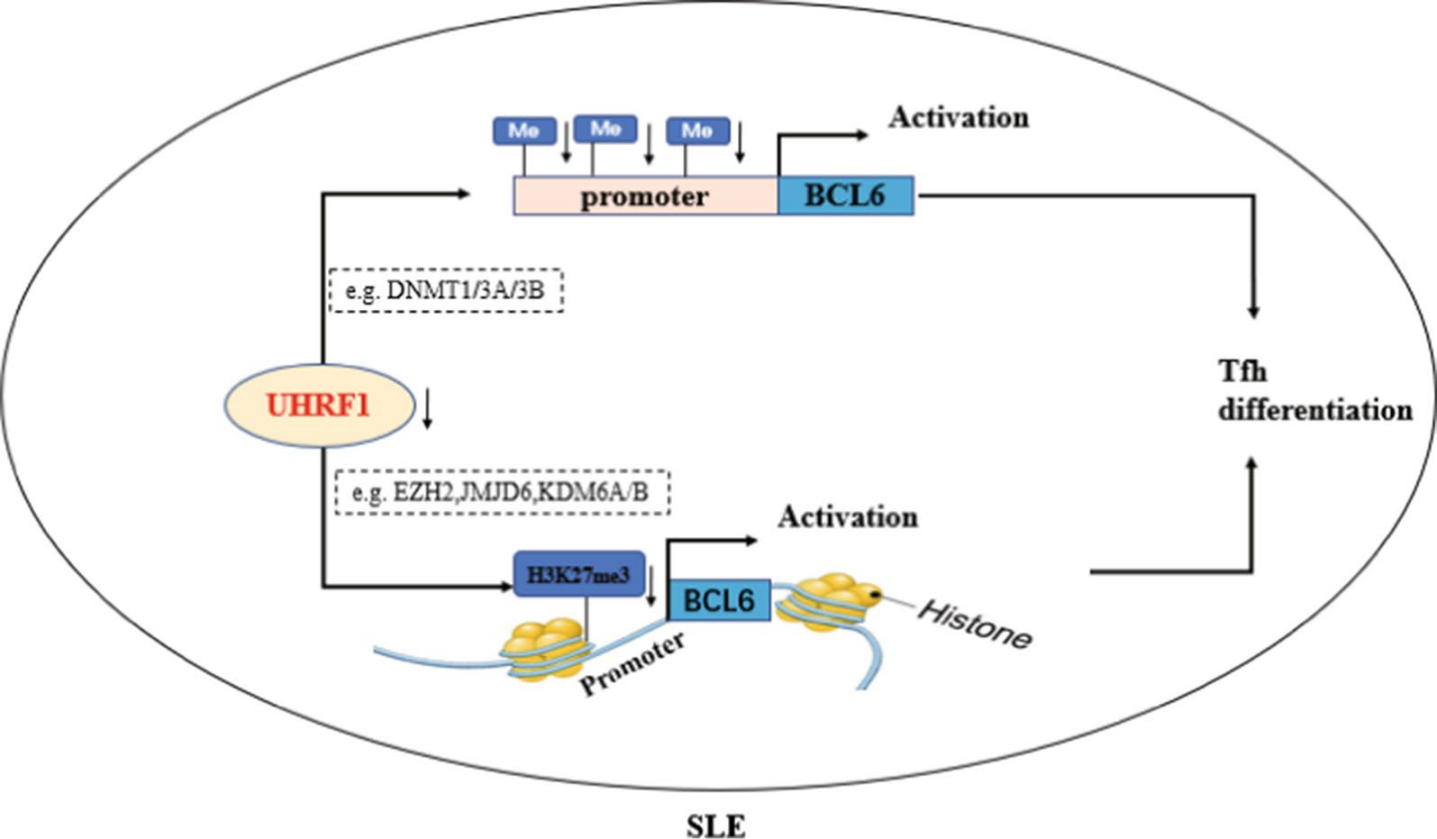
Model of UHRF1 regulates BCL6 in Tfh cells of SLE patients. In SLE patients, UHRF1 downregulation leads to the decreased DNA methylation and H3K27me3, inducing the activation of BCL6 gene transcription to promote Tfh cell differentiation. The potential mechanism of UHRF1 regulating epigenetic modifications in *BCL6* promoter may involve some middle players such as DNMT1, DNMT3A, DNMT3B, EZH2, JMJD6, KDM6A and KDM6B

Previous studies have shown that the epigenetic factor UHRF1 regulates transcription by modulating DNA methylation and histone modification, and plays critical roles in proliferation, development, and tumorigenesis. Recently, some reports indicated that UHRF1 regulates the proliferation, survival, and differentiation of Treg cells and iNKT cells [33–37]. However, whether UHRF1 is involved in regulating the differentiation of Tfh cells and its role in the pathogenesis of SLE still remain unclear. In the present study, we observed that UHRF1 expression in Tfh cells of SLE patients was significantly lower than that of healthy controls. We first found that UHRF1 functions as a repressor in the regulation of BCL6 expression. UHRF1 knockdown could upregulate BCL6 expression by reducing DNA methylation and histone H3K27me3 levels in the BCL6 gene promoter region (Fig. 6). These novel findings suggest that UHRF1 may play an important role in Tfh cells mediated autoimmune diseases.

UHRF1 is abnormally expressed in tumours and immune-related diseases and plays an important role in regulating gene expression and the biological functions of cells. UHRF1 regulates *UBE2L6* gene expression by promoting its hypermethylation in cervical cancer cells to induce apoptosis [38]. UHRF1 is overexpressed in human clear cell renal cell carcinoma and inhibits p53 pathway activation, allowing cells to evade p53-dependent apoptosis [39]. UHRF1 can regulate the methylation levels in the TNF-α gene to enhance the secretion of proinflammatory factors by macrophages [28]. UHRF1 is essential for MIF transcription in macrophages and in B and T lymphocytes [40]. In the present study, we observed that UHRF1 knockdown could promote Tfh cell differentiation, while UHRF1 overexpression had the opposite effects in vitro. We also observed increased levels of Tfh cells, GC-B cells and various antibodies in UHRF1-cKO mice by inducing NP-KLH immunity, indicating that UHRF1 knockout promotes the differentiation of Tfh cells in vivo. These in vitro and in vivo results demonstrated the role of UHRF1 in Tfh cell differentiation and the humoral immune response. In addition, we also observed that UHRF1 had no obvious effect on the cell cycle, apoptosis or proliferation of CD4^+^ T cells (Additional file 1: Figure S1).

A great deal of evidence has shown that aberrant epigenetic modifications contribute to CD4^+^ T cell activation and the pathogenesis of SLE [4, 41–43]. The epigenetic regulator UHRF1 typically alters DNA methylation and histone modification to regulate gene expression. For example, UHRF1 interacts with DNMT1 at the CpG promoter region of Egr1 loci to maintain DNA methylation and affects the nearby histone H3K9me3 and H3K4me3 [31]. UHRF1-cKO in embryonic stem cells may fail to recruit polycomb repressive complex 2 (PRC2) to the promoters of mesodermal genes and shows a reduction of H3K27me3 on mesodermal lineage genes promoters [44]. The results of our previous study showed that the epigenetic modifications of the *BCL6* promoter were altered during Tfh cell differentiation and that BCL6 expression was regulated by the repressive epigenetic modifiers HDAC1 and EZH2 [23]. In the present study, we demonstrated that UHRF1 regulates DNA methylation and H3K27me3 in the BCL6 promoter regions of Tfh cells, suggesting that UHRF1 downregulation promotes Tfh cell differentiation by decreasing the levels of DNA methylation and H3K27me3 in the *BCL6* promoter in SLE. According to previous study, UHRF1 can regulate the epigenetic status of downstream genes through interacting with some epigenetic regulators such as DNMT1, DNMT3A, DNMT3B, EZH2, JMJD6, KDM6A and KDM6B [45], which may be involved in the regulation of BCL6 transcription in Tfh cells of SLE (Fig. 6).

## Conclusions

In summary, the results of our present study demonstrated that UHRF1 downregulation can lead to high BCL6 expression and promote Tfh cell differentiation in vivo and in vitro. This finding reveals the important role of UHRF1 in autoimmune response and provides a potential target for SLE therapy.

## Methods

### Subjects

We recruited 25 SLE patients (22 females and 3 males) from outpatient clinics at the Second Xiangya Hospital of Central South University, fulfilling at least four of the SLE classification criteria of the American College of Rheumatology [46]. Lupus disease activity was assessed using the SLE Disease Activity Index (SLEDAI) [47]. Patient demographic data are shown in Additional file 2: Table S1. 25 Healthy controls (23 females, 2 males) were recruited from the medical staff at the Second Xiangya Hospital, who had no history of cancer, cardiovascular diseases, autoimmune diseases or known infectious diseases. Patients and controls were matched for age and sex. The human sample study followed a protocol approved by the Ethics Committee of Second Xiangya Hospital of Central South University, with written informed consent obtained from the participants.

### Cell isolation and transfection

Venous peripheral blood (100 ml) was drawn from patients and control subjects and preserved with heparin. PBMCs were separated by density gradient centrifugation (GE Healthcare, Switzerland). Total CD4^+^T cells with positive selection and naïve CD4^+^T cell with negative selection were isolated using Miltenyi beads according to the manufacturer’s instructions (Miltenyi, Germany). A density of 1 × 10^6^ PBMCs/mL cultured in RPMI 1640 culture medium (Gibco, CA, USA) supplemented with 10% foetal bovine serum (FBS) in 24-well plates with 2 μl Leukocyte Activation Cocktail (BD Pharmingen™, USA). Total CD4^+^ T cells at a density of 5 × 10^5^ cells/mL were cultured in RPMI 1640 culture medium (Gibco, CA, USA) supplemented with 10% FBS and 2 μg/ml of an anti-CD3 antibody (PeproTech, USA) in 24-well plates precoated and 1 μg/ml of an anti-CD28 antibody (PeproTech, USA). Naïve CD4^+^T cells were transfected with UHRF1-siRNA or Cntl-siRNA (RiboBio Guangzhou, China) using the Human T Nucleofector and Amaxa Nucleofector (Lonza, Switzerland). Then, the cells were seeded and incubated in RPMI 1640 culture medium (Gibco, California, USA) supplemented with 10% FBS and an anti-CD3 antibody (2 μg/ml), anti-CD28 antibody (1 μg/ml) in 24-well plates, recombinant protein IL-6 (20 ng/ml), IL-12 (10 ng/ml), IL-21 (20 ng/ml) and TGF-β (5 ng/ml) for 48 h. The UHRF1 siRNA used in the present study was purchased from RiboBio (China) and had the following sequence: 5′-GGACGAAGTCTTCAAGATT-3’.

### Flow cytometry

Cytokines, transcription factors, and surface markers were evaluated using a FACS Canto II instrument (BD Biosciences, USA). Briefly, for cytokine detection, cells were stimulated for 6 h with Leukocyte Activation Cocktail (BD Pharmingen™, USA). Subsequently, the cells were incubated with FcR blocking reagent (Miltenyi, Germany) for 10 min and then incubated with antibodies against surface markers on ice for 30 min in the dark. For intracellular staining, the cells were fixed and permeabilized with a Transcription Factor Buffer Set (BD Pharmingen™, USA) for 40 min and then stained with fluorescent antibodies for an additional 60 min on ice in the dark. Events were collected and analysed with Tree Star FlowJo. The following antibodies were used in the present study: UHRF1 (Abcam, UK), goat anti-Rb IgG Alexa Fluor® 488 (Abcam, UK), PE-cy7 anti-human CD3 (BD Pharmingen™, USA), APC-cy7 anti-human CD4 (BD Pharmingen™, USA), APC anti-human bcl-6 (BD Pharmingen™, USA), FITC anti-human CD4 (BD Pharmingen™, USA), PE-cy7 anti-human PD1 (BD Pharmingen™, USA), PerCP-Cy5.5 anti-human CXCR5 (BD Pharmingen™, USA). Ms CD45R/B220 FITC RA3-6B2 (BD Pharmingen™, USA), Ms T/B-cell antigen Alexa 647 GL7 (BD Pharmingen™, USA), PE/Cy7 anti-human CD95 (Fas) (Biolegend, USA), Ms CXCR5 biotin 2G8 100 μg (BD Pharmingen™, USA), streptavidin-PE 500 μg (BD Pharmingen™, USA), and anti-mouse CD279 (PD-1) (J43) APC-EFLUOR (eBioscience, USA).

### ELISA

NP-KLH-specific antibodies were measured with 10 μg/ml of NP-BSA (Bioresearch Technologies) as the coating reagent for ELISA. Diluted serum was incubated in the wells for 1 h at room temperature. Then, NP-KLH-specific antibodies (IgG, IgG1, IgG2a, IgG2b and IgM) were detected using goat polyclonal anti-IgG HRP (Southern Biotech), anti-IgG1 HRP (Southern Biotech), anti-IgG2a HRP (Southern Biotech), anti-IgG2bHRP (Southern Biotech), and anti-IgM HRP (Southern Biotech).

### MeDIP-qPCR

UHRF1-siRNA or UHRF1 lentiviral expression vectors (UHRF1-lentivirus) were transfected into naïve CD4^+^T cells. Genomic DNA was extracted from cells using a Qiagen DNA Extraction kit following the manufacturer’s instructions (QIAGEN, Germany). MeDIP analysis was performed according to the manufacturer’s instructions provided in the MeDIP assay kit (Active Motif, CA, USA). Precipitated DNA was amplified by quantitative PCR using forward and reverse primers specific to the BCL6 promoter sequence (Additional file 3: Table S2).

### Immunofluorescence

For tissue samples, mesenteric lymph nodes (mLNs) were fixed with formalin and embedded with paraffin. The following antibodies were used for immunofluorescence analysis: anti-peanut agglutinin (anti-PNA, 20 μg/ml, Vector Laboratories), an anti-goat HRP-linked antibody (Abcam, UK), and an anti-CD3 antibody (Abcam, UK). Images were obtained using a laser scanning microscope (Olympus, Japan).

### RNA isolation and real-time quantitative polymerase chain reaction

Naïve CD4^+^T cells, transfected with UHRF1-siRNA or Cntl-siRNA for 48 h and UHRF1-lentivirus or Cntl-lentivirus, were lysed with TRIzol reagent (Invitrogen, USA) according to the manufacturer’s instructions. Real-time quantitative polymerase chain reaction (RT-qPCR) was performed using a LightCycler 96 (Roche, Basel, Switzerland), and the mRNA or DNA levels were quantified using a SYBR Prime Script RT-qPCR kit (Takara, Dalian, China). β-Actin was amplified and used as a loading control. The relative mRNA or DNA levels were calculated using the 2 ^−ΔCt^ (ΔCt = Ct _target gene_—Ct _β-actin_) method. The sequences of the primers are shown in Additional file 3: Table S2.

### Western blot analysis

Cells were lysed with whole cell lysis buffer and denatured at 100 °C for 5 min. Subsequently, the cellular proteins were separated by SDS polyacrylamide gel electrophoresis and transferred to PVDF membranes. The membranes were blocked in TBST buffer that contained 5% non-fat dry milk and then incubated overnight at 4 ℃ with a rabbit anti-UHRF1 Ab (1:1000, Abcam, UK) and a rabbit anti-β-actin Ab (1:1000, Abcam, UK).

### Chromatin immunoprecipitation (ChIP)-qPCR

ChIP was performed according to the instructions provided with ChIP assay kit (Millipore, Billerica, MA, USA). In brief, Naïve CD4^+^ T cells transfected with UHRF1-siRNA or Cntl-siRNA, UHRF1-lentivirus or Cntl-lentivirus were harvested and fixed for 10 min at RT with 1% formaldehyde. Glycine was subsequently added to a final concentration of 0.125 M to quench the formaldehyde. Cells were pelleted, washed once with ice-cold PBS, and lysed with SDS buffer. Lysates were pelleted, resuspended, and sonicated to reduce DNA to fragments of 200 to 1000 base pairs. Chromatin was precipitated with protein A agarose beads for 1 h and then incubated with 5 μl anti-acetyl histone H3 (Active Motif), or 5 μl anti-H3K27me3 (Active Motif) or normal IgG (Millipore) overnight. The immunocomplexes were further precipitated with protein A agarose beads, washed, and eluted in 100 ml of TE with 0.5% SDS and 200 mg/ml proteinase K. Precipitated DNA was further purified with phenol/chloroform extraction and ethanol. The relative enrichment level was quantified using qPCR and calculated relative to the respective input DNA. The primers are shown in Additional file 3: Table S2.

### Lentivirus transfection

UHRF1-lentivirus and Cntl-lentivirus were packaged and synthesized by Genechem Biotechnology (Genechem, Shanghai, China). We infected 0.5 × 10^6^ naïve CD4^+^T cells at an appropriate multiplicity of infection (MOI = 30) for 12 h, after which the cells were seeded and incubated in RPMI 1640 culture medium (Gibco, CA, USA) supplemented with 10% FBS and anti-CD3 antibody (2 μg/ml) and anti-CD28 antibody (1 μg/ml) in 24-well plates and then treated with recombinant IL-6 (20 ng/ml), IL-12 (10 ng/ml), IL-21 (20 ng/ml) and TGF-β (5 ng/ml) for 72 h.

### NP-KLH immunization

Age-matched mice were immunized with NP-KLH (Bioresearch Technologies, USA) emulsified in Imject™ Alum Adjuvant (Thermo Fisher, USA) that was administered to each mouse by subcutaneous injection. After immunization for one week, we injected the same amount of NP-LKH to boost mouse immunity for another week. Then, we collected the spleen, lymph nodes and serum for subsequent experiments.

### Cell cycle analysis

Naïve CD4^+^ T cells transfected with UHRF1-siRNA or Cntl-siRNA for 96 h were harvested. Cells were fixed in 75% ethanol under −20 ℃ overnight and washed by PBS, then cells were added with 500 μl PI staining solution and incubated for 15 min in the dark at room temperature. All cells were evaluated using a FACS Canto II instrument (BD Biosciences, USA) and the dates were analyzed with FlowJo software (Tree Star FlowJo).

### Apoptosis assays

Naïve CD4^+^T cells transfected with UHRF1-siRNA and Cntl-siRNA for 96 h were harvested. Apoptotic cell populations were detected by a FACS Canto II instrument (BD Biosciences, USA) using the Annexin V-FITC Apoptosis Kit (Roche, Basel, Switzerland) according to the manufacturer’s instruction.

### Proliferation assay

Briefly, 1 × 10 ^4^ UHRF1-siRNA or Cntl-siRNA naïve CD4^+^ T cells were transferred into a 96-well cell culture plate with 100 μl the RPMI 1640 medium and cultured for 96 h. Later on, 10 μl CCK-8 was added to each well, and then the plates were incubated for 2 h. Eventually, absorbance was measured at 450 nm with a microplate reader (BioRad Laboratories, CA, USA).

### Statistical analysis

Data are presented as the means ± standard deviation (S.D.). We used an unpaired two-tailed *t*-test to compare the difference between two groups. All analyses were performed with SPSS 19.0 (SPSS, Inc., Chicago, IL). *P* < 0.05 was considered to indicate a significant difference.

## Supplementary information

**Additional file 1:** **Figure S1**. UHRF1 knockdown had no effect on cell cycle (a), apoptosis (b) and cell proliferation (c) in cells with UHRF1-siRNA compared to the negative control. The values are the averages of at three biological replicates, and all data are shown the means ± SD. *P < 0.05 relative to control.

**Additional file 2: Table S1**. Patient demographics.

**Additional file 3: Table S2**. primers for qPCR.

**Additional file 4**. Raw images of gels or blots.

## Data Availability

All data is available from the corresponding author on reasonable request.

## Abbreviations

SLE: Systemic lupus erythematosus
GC: Germinal center
UHRF1: Ubiquitin-like with PHD and RING Finger domains 1
PD1: Programmed cell death-1
ICOS: Inducible T-cell costimulatory
FCM: Flow cytometry
MFI: Mean fluorescence intensity
FBS: Fetal bovine serum
PBMCs: Peripheral blood mononuclear cells
H3K27me3: H3K27 tri-methylation
ChIP: Chromatin immunoprecipitation
RT-qPCR: Real-time quantitative polymerase chain reaction
NP-KLH: NP-Keyhole Limpet Hemocyanin
PNA: Peanut Agglutinin
DLNs: Draining lymph nodes
DNMT1: DNA methyltransferase 1
