# Supplementary figures and images for: UHRF1 downregulation promotes T follicular helper cell differentiation by increasing BCL6 expression in SLE
Source: Clin Epigenetics. 2021 Feb 10;13:31. doi: 10.1186/s13148-021-01007-7 (PMC7874639; doi:10.1186/s13148-021-01007-7)

a

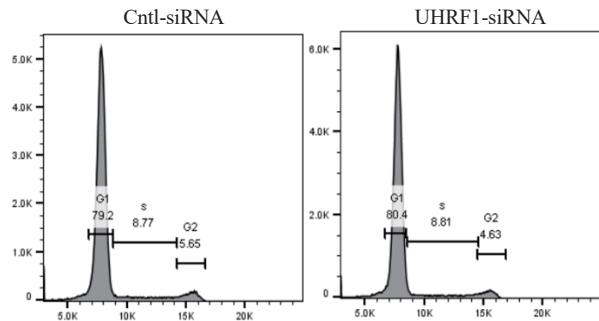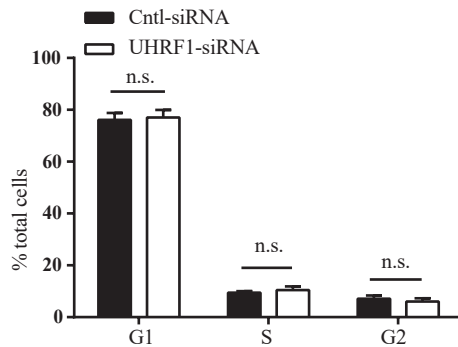

b

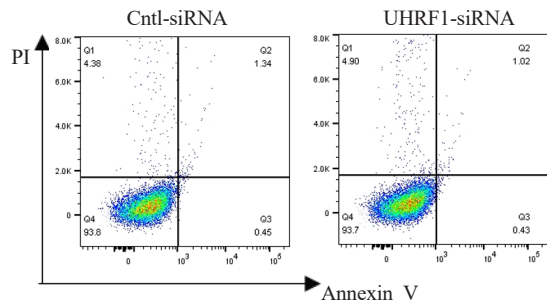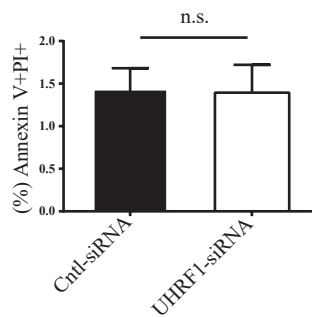

c

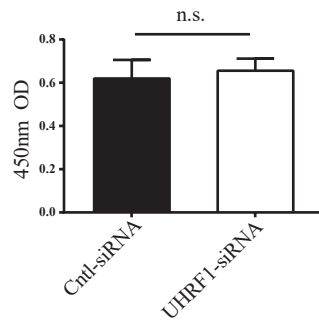

Supplement: Supplementary file 1 — Additional file 1: Figure S1. UHRF1 knockdown had no effect on cell cycle (a), apoptosis (b) and cell proliferation (c) in cells with UHRF1-siRNA compared to the negative control. The values are the averages of at three biological replicates, and all data are shown the means ± SD. *P < 0.05 relative to control. [file 13148_2021_1007_MOESM1_ESM.pdf]
