# Supplementary material for: UHRF1 downregulation promotes T follicular helper cell differentiation by increasing BCL6 expression in SLE
Source: Clin Epigenetics. 2021 Feb 10;13:31. doi: 10.1186/s13148-021-01007-7 (PMC7874639; doi:10.1186/s13148-021-01007-7)
Supplement: Supplementary file 2 — Additional file 2: Table S1. Patient demographics. [file 13148_2021_1007_MOESM2_ESM.doc]

Table S1. Patient demographics

| Patients | SLEDAI | Age (years) | Gender | Smoking |
| --- | --- | --- | --- | --- |
| 1 | 5 | 51 | F | No |
| 2 | 6 | 20 | F | No |
| 3 | 6 | 22 | F | No |
| 4 | 5 | 51 | F | No |
| 5 | 12 | 35 | F | No |
| 6 | 9 | 42 | F | No |
| 7 | 8 | 44 | F | No |
| 8 | 2 | 32 | M | No |
| 9 | 2 | 33 | F | No |
| 10 | 4 | 28 | F | No |
| 11 | 2 | 72 | F | No |
| 12 | 0 | 57 | F | No |
| 13 | 8 | 24 | F | No |
| 14 | 7 | 61 | F | No |
| 15 | 2 | 17 | M | No |
| 16 | 8 | 29 | F | No |
| 17 | 6 | 40 | F | No |
| 18 | 18 | 34 | F | No |
| 19 | 4 | 54 | M | No |
| 20 | 8 | 15 | F | No |
| 21 | 4 | 32 | F | No |
| 22 | 2 | 42 | F | No |
| 23 | 8 | 25 | F | No |
| 24 | 12 | 38 | F | No |
| 25 | 6 | 56 | F | No |

SLEDAI: Systemic Lupus Erythematosus Disease Activity Index, F: female, M: Male,
