# Supplementary material for: UHRF1 downregulation promotes T follicular helper cell differentiation by increasing BCL6 expression in SLE
Source: Clin Epigenetics. 2021 Feb 10;13:31. doi: 10.1186/s13148-021-01007-7 (PMC7874639; doi:10.1186/s13148-021-01007-7)
Supplement: Supplementary file 3 — Additional file 3: Table S2. primers for qPCR. [file 13148_2021_1007_MOESM3_ESM.docx]

Table S2: primers for qPCR

| *BCL6* promoter region 1 (P1): | forward | 5′-AGCTGCTTCTGGTCCAAACAT-3′ |
| --- | --- | --- |
|  | reverse | 5′-CAATGCACTACACGCAGCAC-3′ |
| *BCL6* promoter region 2 (P2) | forward | 5′-TAAACCCACCACTTGCTGTCC-3′ |
|  | reverse | 5′-TCACAGGATCGGCATCGGT-3′ |
| *BCL6* promoter region 3 (P3) | forward | 5′-CTCTTACTCGCCTCTCTAACCC-3′ |
|  | reverse | 5′- CTGTAGCAAAGCTCGGCCTC-3′ |
| β-actin（NM_001101.5） | forward | 5′-CATGTACGTTGCTATCCAGGC-3′ |
|  | reverse | 5′- CTCCTTAATGTCACGCACGAT-3′ |
| UHRF1（NM_001290050.2） | forward | 5′-TGCTCCCATCAATGACCAAG-3′ |
|  | reverse | 5′-GGGCCAGTATTTCACAACCT-3′ |
| BCL6（NM_001130845.2） | forward | 5′- CTCCCATGTGTCTTCAGCTTTC -3′ |
|  | reverse | 5′- TCGGCTCCAAGGTTAGTGTG -3′ |
