# Supplementary material for: UHRF1 downregulation promotes T follicular helper cell differentiation by increasing BCL6 expression in SLE
Source: Clin Epigenetics. 2021 Feb 10;13:31. doi: 10.1186/s13148-021-01007-7 (PMC7874639; doi:10.1186/s13148-021-01007-7)
Supplement: Supplementary file 4 — Additional file 4. Raw images of gels or blots. [file 13148_2021_1007_MOESM4_ESM.doc]

1，blot-si-UHRF1（fugure2,b）


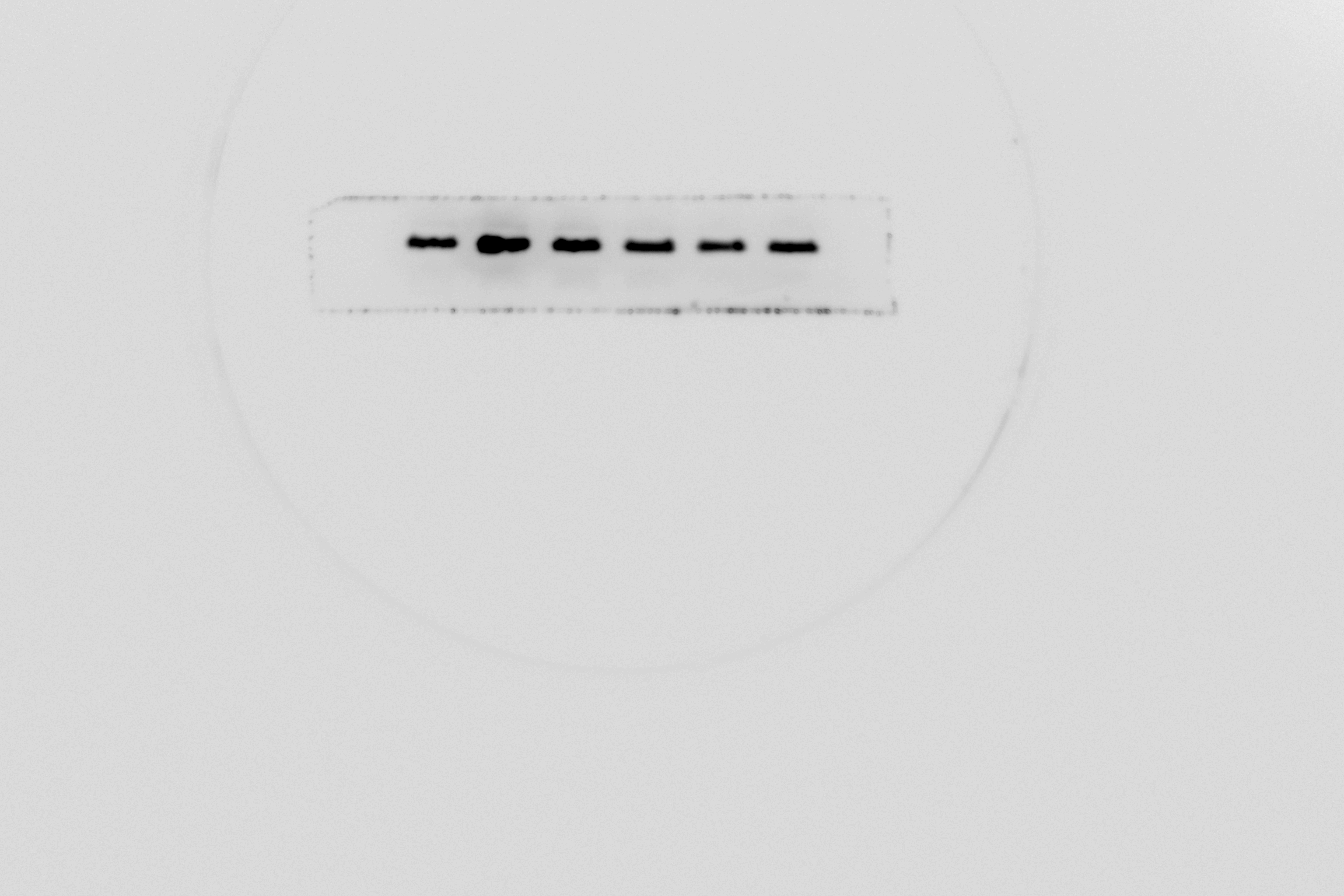


Cntl UHRF1-siRNA Cntl UHRF1-siRNA Cntl UHRF1-siRNA


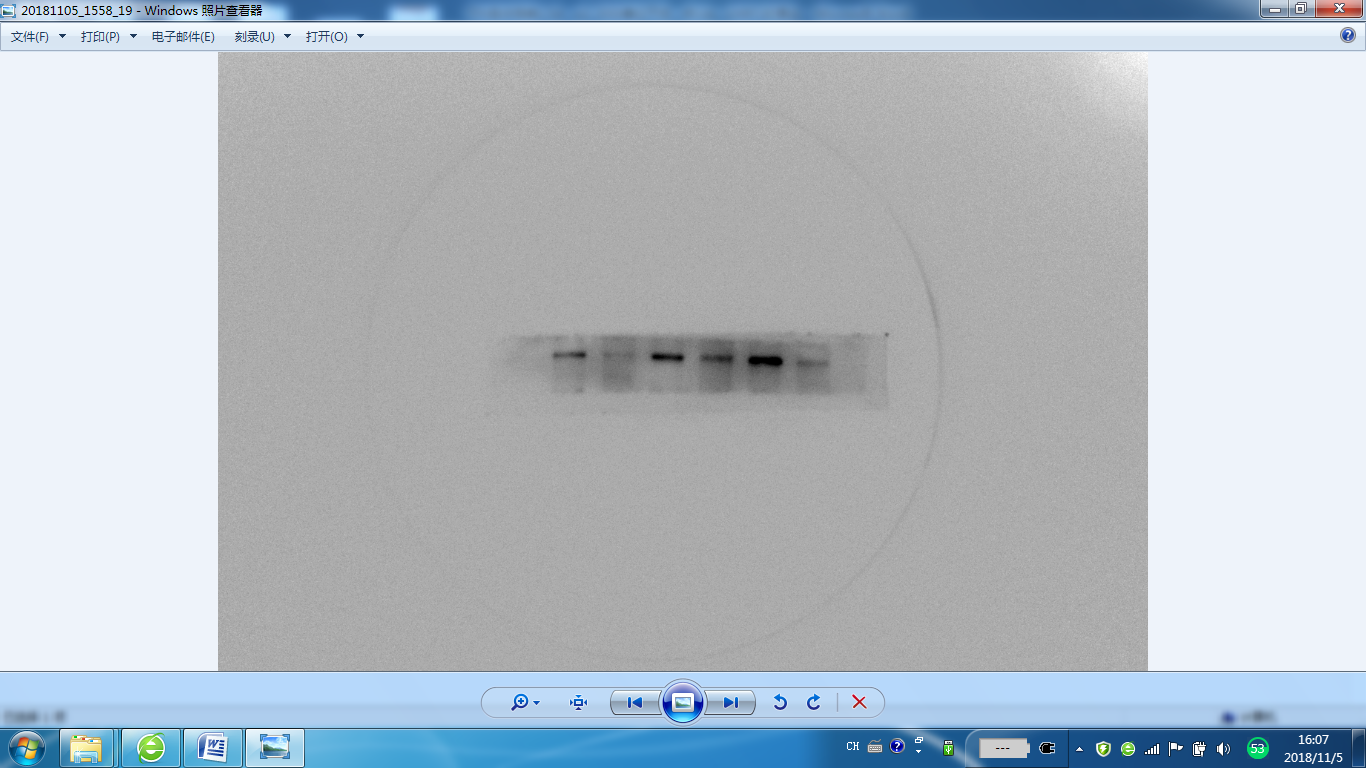


UHRF1:95kd

Actin:43kd

2，blot-lenti-UHRF1（figure3.b）

95kd UHRF1

43kd actin


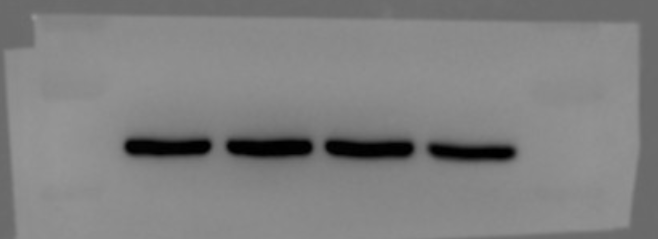


Cntl-letirirus Cntl-letirirus UHRF1-lentivirus UHRF1-lentivirus


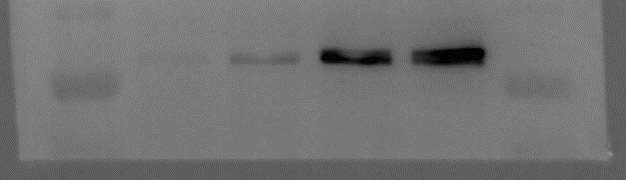


3,gel(fugure5a)

UHRF1 cre

**f/f f/+ +/+ - +**


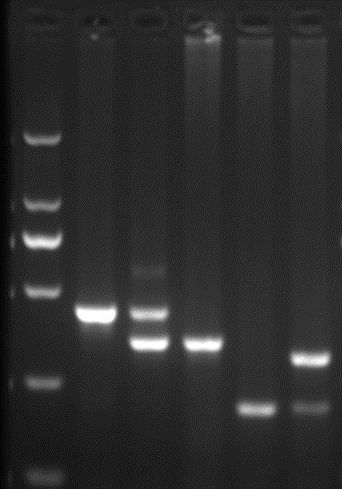


1000

2000

750

500

250

100

4, blot (fugure5b)

Wt UHRF1-cko Wt UHRF1-cko


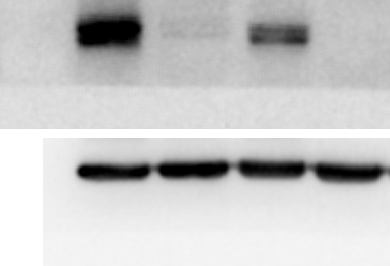


95kd UHRF1

43kd actin
